# How Strong Can We Pull? Critical Thresholds for Traction Forces on the Aortic Annulus: Measurements on Fresh Porcine Hearts

**DOI:** 10.3390/medicina58081055

**Published:** 2022-08-04

**Authors:** Martin Hartrumpf, Josephine Sterner, Filip Schroeter, Ralf-Uwe Kuehnel, Roya Ostovar, Johannes M. Albes

**Affiliations:** Department of Cardiovascular Surgery, Heart Center Brandenburg, University Hospital Brandenburg Medical School (Theodor Fontane), Ladeburger Strasse 17, 16321 Bernau bei Berlin, Germany

**Keywords:** aortic valve, valve surgery, valve prosthesis, surgical technique, tourniquet, experimental surgery

## Abstract

*Background and Objectives:* Friable or infected tissue remains a challenge in surgical aortic valve replacement. We recently described the “Caput medusae” method, in which circumferential tourniquets temporarily secure the prosthesis and are then gently knotted. Tourniquets have been shown to develop significantly less force than knots. The current study investigates the critical threshold forces for tissue damage to the aortic annulus. *Materials and Methods:* In 14 fresh porcine hearts, the aortic valve leaflets were removed and several pledgeted sutures were placed along the annulus at defined locations. The hearts were mounted in a self-constructed device. Incremental traction force was applied to every suture and continuously recorded. The movement of each Teflon pledget was filmed with a high-speed camera. Forces at the moment of pledget “cut-in” as well as complete “tear-out” were determined from the recordings. *Results:* The average threshold force was determined 9.31 ± 6.04 N for cut-in and 20.41 ± 10.02 N for tear-out. Detailed analysis showed that the right coronary region had lower threshold forces than the other regions (4.77 ± 3.28 N (range, 1.67–12.75 N) vs. 10.67 ± 6.04 N (1.62–26.00 N) for cut-in and 10.67 ± 4.04 N (5.40–18.64 N) vs. 23.33 ± 9.42 N (9.22–51.23 N) for tear-out). The findings are discussed in conjunction with the knot and tourniquet forces from our previous study. *Conclusions:* Even in healthy tissue, moderate forces can reach a critical level at which a Teflon pledget will cut into the annulus, while a complete tear-out is unlikely. The right coronary portion is more susceptible to damage than the remaining regions. When compared to previous data, forces during manual knotting may exceed the critical cut-in level, while rubber tourniquets may provide a higher safety margin against tissue rupture.

## 1. Introduction

Friable or infected tissue remains a challenge in surgical aortic valve replacement. Tearing of the native annulus or rupture of a suture can cause paravalvular leakage, which is known to affect long-term survival if uncorrected [1,2,3]. Various techniques, including rubber tourniquets, have been developed to address this problem and to facilitate insertion of the prosthetic valve. Temporary fixation has been suggested when a small annulus or an obstructing subvalvular structure is present [4]. In several instances, annular enlargement could be avoided in this way. In the case of endocarditis, rubber tourniquets can be used for closure of abscess cavities [5]. They may also be helpful in mitral or tricuspid valve repair. However, most authors apply the tourniquet technique discontinuously. For better prevention of paravalvular leakage in aortic valve endocarditis, we have introduced continuous placement of rubber tourniquets along the annular circumference and named them “Caput medusae” due to their appearance in reference to Greek mythology [6]. The tourniquets are then replaced with gentle knots, one at a time. Under the protection of the snares, this can be accomplished with much less force than usual.

To quantify the forces involved in these maneuvers, we had previously performed a volunteer study with our cardiosurgical staff. In an experimental setup, the average forces of traditional suture tying versus tightening of rubber tourniquets were determined in a series of 18 staff surgeons [7]. Here, we demonstrated that singular tourniquets exert a local force of 1.08 ± 0.48 N compared with classical knotting, which reaches forces of up to 13.64 ± 5.76 N.

The current study complements our previous work and focuses on the effects of the generated forces on the native tissue. In particular, it aims to determine the thresholds for the forces required to cause rupture of the aortic annulus in a series of fresh porcine hearts, including their position. To our knowledge, this has not been quantified regionally in the aortic annulus. There are only few force measurements on the porcine ascending aorta [8], mitral annulus [9], mitral/tricuspid annulus [10], and mitral/aortic annulus [11]. The results and their clinical significance are discussed in conjunction with our previous data.

## 2. Materials and Methods

### 2.1. Study Design and Technical Setup

The study was conducted in a laboratory setting with the exclusive use of isolated porcine hearts. No patients were involved. Therefore, ethics committee approval was not required.

Porcine hearts were regularly purchased from a local slaughterhouse in compliance with the applicable hygiene regulations. They were obtained in several batches one day before the experiments and stored at 5 °C to keep them fresh until final use. In accordance with government regulations, a small incision must be made in the left ventricle during collection. This did not affect our measurements.

**Figure 1:**
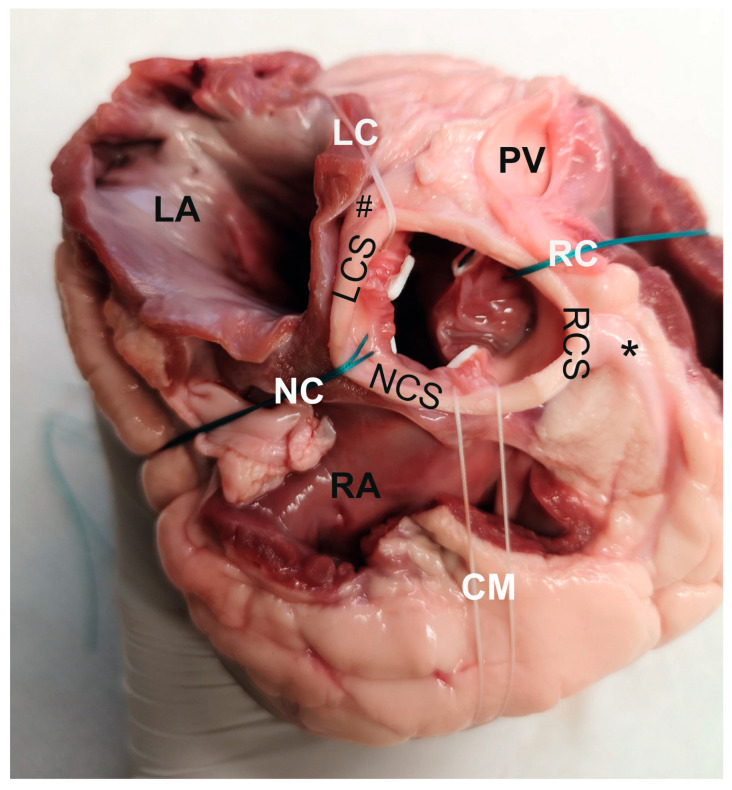
Example of a porcine heart from the top view with the aorta removed and four sutures in place. #, Left coronary artery; *, Right coronary artery; CM, commissural suture (here: between right and noncoronary leaflet); LA, left atrium (cut); LC, left coronary suture; LCS, left coronary sinus; NC, noncoronary suture; NCS, noncoronary sinus; PV, pulmonary valve (cut); RA, right atrium (cut); RC, right coronary suture; RCS, right coronary sinus.

The aortic root was dissected free and the cusps carefully removed. 3 to 4 pledgeted valve sutures (Ethibond Excel™ 2-0, Ethicon, Johnson & Johnson Medical GmbH, Norderstedt, Germany) were subsequently placed along the native aortic annulus as if a supraannular valve had been implanted in a standard manner. Care was taken to include (I) the right, (II) the left, and (III) the noncoronary portion of the circumference and (IV) one of the commissures (Figure 1).

**Figure 2:**
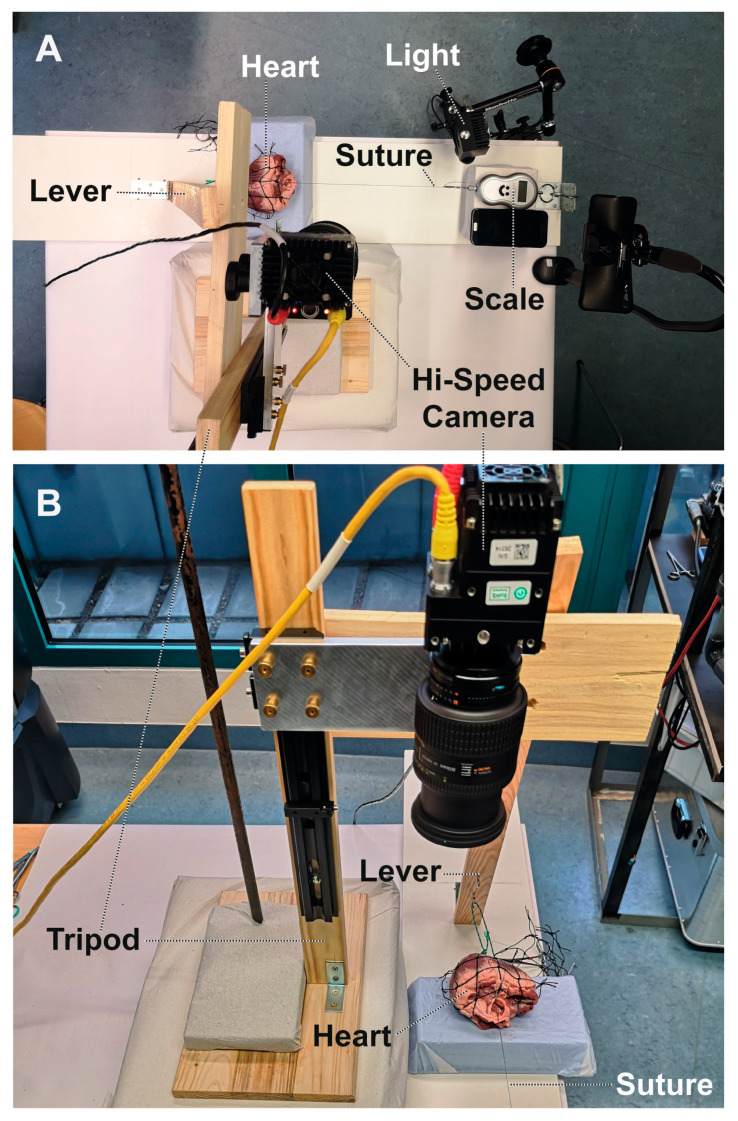
Experimental setup. (**A**) Top view. The heart was mounted in a ‘stretching bench’ where increasing traction force could be applied to a single pledgeted annular suture. Force was generated by slowly pulling a lever and measured with a luggage scale. (**B**) Front view. The motion of the respective Teflon pledget was continuously filmed with a high-speed camera at 1000 frames per second and recorded for offline analysis. The entire procedure was repeated for each suture and for all hearts one after another.

Each heart was then attached to a self-constructed device that served as a “stretching bench” (Figure 2A). For this purpose, the heart was clamped in a plastic mesh and attached to the base plate. An increasing force was applied to each suture, one by one, by slowly pulling a 1:5 lever to which the suture was attached. The traction forces were measured continuously with a luggage scale (up to 40 kg, accuracy 10 g), which was set to zero before each run. The lever was pulled slowly until complete tear-out of the suture occurred. The motion of each subannular Teflon™ pledget was recorded with a high-speed camera (Phantom Miro C320, High Speed Vision GmbH, Ettlingen, Germany) at 1000 fps (Figure 2B). At the same time, the luggage scale was continuously filmed with a smartphone at 30 fps to store the force values shown on the scale display (time resolution, 0.125 s). Accurate time synchronization could be ensured by taking into account the time stamps of both recordings. The current suture position in the annulus was also recorded.

**Figure 3:**
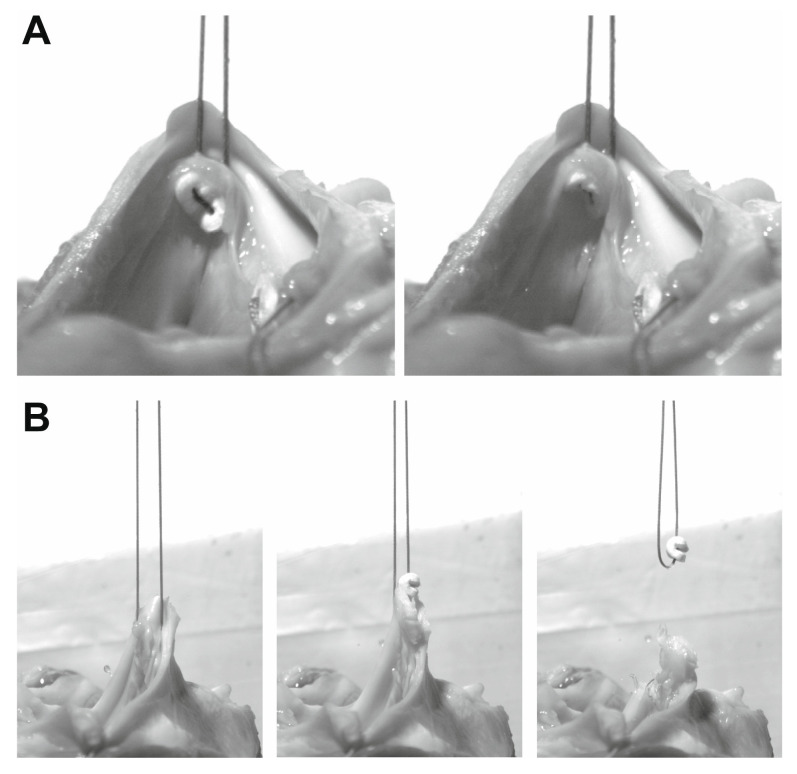
Representative illustration of pledget behavior under traction. Still images were taken from the high-speed recordings. (**A**) Moment of “cut-in”. As soon as the critical force is reached, the subannular pledget suddenly migrates into the tissue. (**B**) Moment of “tear-out”. At a more distant force level, the annulus tears and releases the pledget with a jerk.

During the study, it became apparent that the tear-out of a pledget seems to be a two-step process: we could first observe a sudden migration of the pledget into the tissue, called “cut-in” (Figure 3A). When the pulling force continued to increase, a complete “tear-out” occurred at a later stage (Figure 3B). The respective force values were recorded separately for both events.

Offline analysis was carried out at a later stage according to the following algorithm: the high-speed recording represented the guiding instrument due to the highest time resolution. This tool was used to determine the exact time of the index event (i.e., cut-in or tear-out), although few cases remained indeterminable. Complete tearing was easier to detect and corresponded to the highest value on the scale display. Once the exact time was determined, we searched for the corresponding timestamp on the smartphone video and immediately read the corresponding scale value from the recording. We did not attempt to extrapolate any values as the increase in force was not linear but showed a progressive slope when approaching the final tear-out. To ensure comparability of the measurements, we strictly adhered to this scheme throughout.

### 2.2. Statistical Analysis

The display values of the luggage scale were converted from (kg) to (N) by multiplying with 9.81 m/s^2^. Statistics were calculated using IBM SPSS 23 (IBM, Armonk, NY, USA). Average force values were computed per anatomic position to compensate for the variance among the hearts and displayed as mean ± SD and median. Corresponding boxplots were created. Normality was confirmed by Kolmogorov–Smirnov tests. A non-nested two-factor design was applied to both types of force. The ID number of the heart was treated as a random factor, while the suture position within the annulus was declared a fixed factor. Univariable ANOVA was conducted for both forces relative to the suture position using a main-effects model. Post-hoc tests were generated to account for possible differences as to the anatomic location. In particular, Tukey’s HSD and Student–Newman–Keuls test were applied. The measured forces were finally plotted on an overall scale, together with the knotting and tourniquet forces we had determined earlier.

## 3. Results

A total of 14 healthy porcine hearts were used. For the right, left, noncoronary, and commissural positions, there were 12, 14, 13, and 13 valid cut-in measurements and 13, 14, 13, and 15 tear-out measurements. The variation is explained by differences in tissue suitability and by a slightly less reliable determination of the cut-ins. Force values were normally distributed for all regions. The corrected results of the Kolmogorov–Smirnov test for the right, left, noncoronary, and commissural regions were *p* = 0.114, 0.200, 0.200, 0.200 for the cut-in values and *p* = 0.105, 0.200, 0.200 for the tear-out values, respectively.

**Figure 4:**
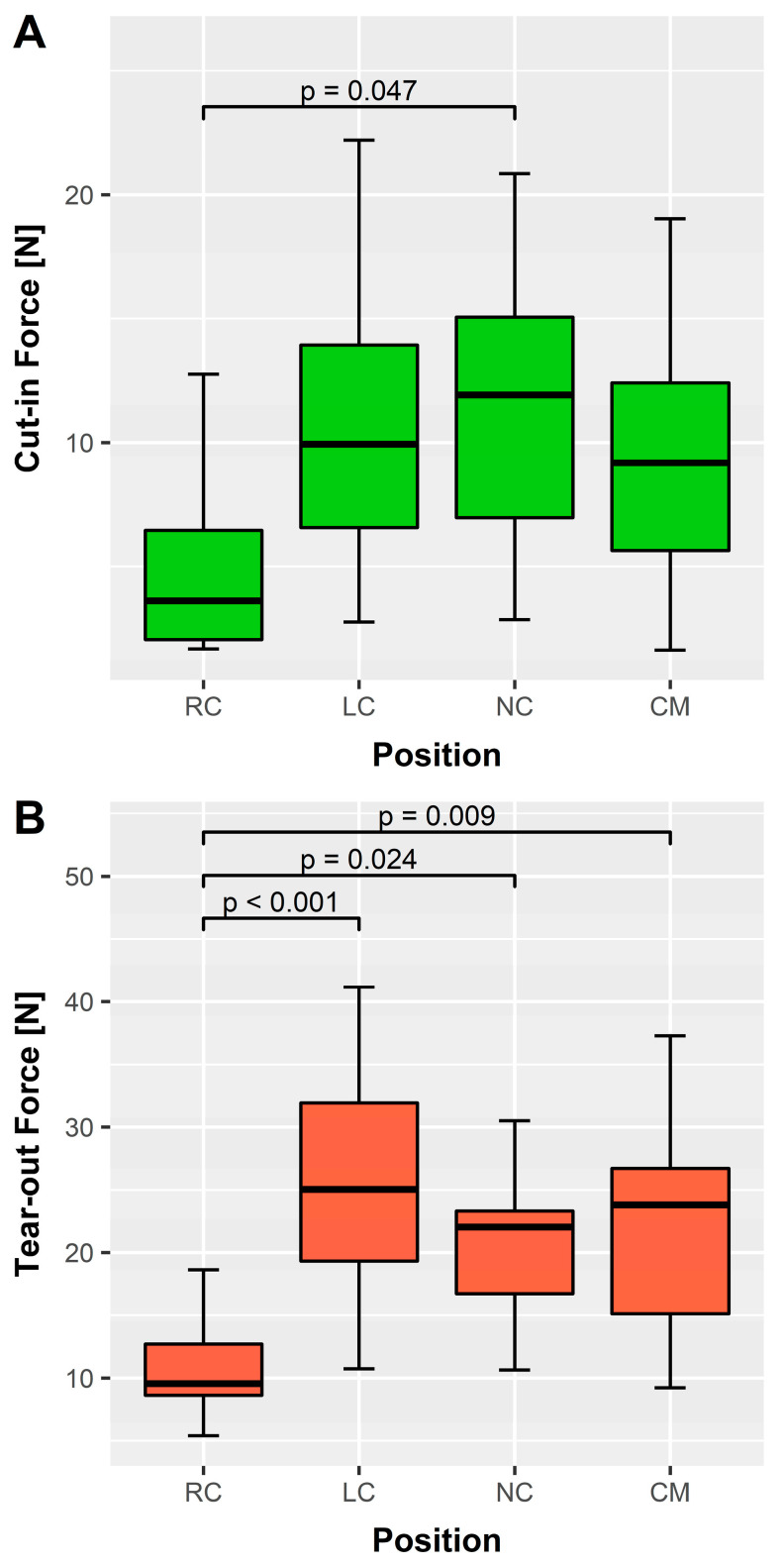
Boxplots showing the threshold forces in relation to the suture position. (**A**) Cut-in forces (green); (**B**) Tear-out forces (red). The right coronary position shows the lowest forces necessary to cause damage. Additionally, indicated are the significant differences (5% level) according to Tukey’s post-hoc test, including their *p* values. CM, commissure; LC, left coronary; NC, noncoronary; RC, right coronary.

The overall traction force was 9.31 ± 6.04 N for cut-in and 20.41 ± 10.02 N for tear-out. Table 1 and Figure 4 show the average traction forces arranged according to their anatomic location. The combined mean force for the left coronary, noncoronary, and commissural regions was 10.67 ± 6.04 N (range, 1.62–26.00 N) to cause a cut-in and 23.33 ± 9.42 N (9.22–51.23 N) to cause a complete tear-out. In contrast, the forces acting on the right coronary region were much lower to produce the same effect. They were less than half of the other forces (see Table 1).

To further decompose the results according to possible influence factors, we employed a two-factorial ANOVA model (Table 2). The identity of the heart itself has no systematic impact on the cut-in or tear-out forces (*p* = 0.724 and *p* = 0.611, respectively). In contrast, there is a significant effect of the anatomic location of the suture. As suggested in Figure 4, the significant deviation is caused by the lower force levels for the right coronary region compared to the other regions.

Post-hoc analysis confirmed significant differences between the right coronary region and the other regions in terms of tear-out forces (Figure 4B). There was a significant difference between right and noncoronary regions for the cut-in forces (Figure 4A). The Student–Newman–Keuls test yielded two homogeneous subsets for the mean values. One subset comprised the commissures, left, and noncoronary regions (*p* = 0.912 for cut-in, *p* = 0.214 for tear-out), while the second subset was represented by the lower values of the right coronary region (see also Table S1 Raw Data).

## 4. Discussion

Implantation of a prosthetic aortic valve is part of daily practice in the operating room, where we do many things subconsciously. No one thinks about tying the knots and the forces that occur. We know from experience how to adjust the strength of a knot to the particular annulus. However, we do not really know how far we are from the load limit of the tissue. Especially in elderly and frail patients, or in those with endocarditis, we are often faced with fragile tissue that does not withstand the forces of tying the knots well. Laceration of the tissue, or even a complete tearing of the suture, may occur, resulting in detachment of the prosthesis with consecutive paravalvular leakage. A previous study has shown that the surgeon themselves are among the predictive factors for paravalvular leakage [2]. Likewise, an ovine model of mitral annuloplasty showed that different tie-down forces occur depending on the surgeon and the suture position [12]. This raises further questions about the relevance of technical skills, personal attitude, and surgical training, all of which can influence the clinical outcome [13].

To account for the reduced tissue quality, we routinely use circular pre-fixation of the prosthesis with a series of rubber tourniquets called “Caput medusae” at our institution [6]. From earlier measurements, we have learned that a single tourniquet exerts a force 13 to 14 times lower than the maximum force of a single surgical knot [7]. Thus, the tourniquet technique is gentler on the tissue and allows for much smoother subsequent knotting.

The current study complements our previous work. We have attempted to determine the thresholds for forces leading to damage of the aortic annulus using porcine hearts. Such hearts are easy to obtain and closely resemble the human heart in size and anatomy [14]. In a first approach, we did not intend to simulate a model with friable tissue, but used healthy hearts instead. To keep all tissue properties as realistic as possible, we refrained from using preserved or deep-frozen specimens. To account for natural variation, we examined a number of porcine hearts and placed the sutures at different locations per heart.

During the experiments, it became apparent that the damage to the annulus occurs in two stages. Upon reaching a first critical force level, the Teflon™ pledget suddenly cuts into the annular tissue (Figure 3A). This event is known from the operating room when there is a sudden loss of resistance and the pledget unexpectedly disappears from the otherwise closed row. It does not necessarily lead to paravalvular leakage, but it definitely creates a weak spot. As the force continues to increase, the tissue becomes progressively stretched and finally a tear-out occurs (Figure 3B). However, force values in this case are more than twice as high (20.41 ± 10.02 vs. 9.31 ± 6.04 N) and are normally not reached, even when a suture is tied firmly.

Likewise, this two-step process was also observed by other authors [11]. Once the suture is fully tensioned, the tissue behaves elastically until the “yield point” is reached, where tearing begins. This corresponds to our “cut-in” point. Complete rupture occurs at a variably higher force corresponding to our “tear-out” level. The authors determined the respective forces to be 4.0 ± 3.3 N (range, 0.2–14.0 N) and 4.9 ± 3.6 N (range, 1.4–14.0 N), which is within a much lower range than we observed. However, these samples were obtained intraoperatively from diseased patients during aortic or mitral valve replacement and were subsequently preserved and frozen. These results are therefore not comparable with our findings.

Compared with our previous data, one of the main findings is that the average knotting force, determined to be 13.64 ± 5.88 N [7], exceeds the current cut-in level. This suggests that knot tying already falls into the critical range and may expose the patient to the risk of paravalvular leakage. If the surgeon’s force is not carefully adjusted to the patient’s individual tissue, a vulnerable spot may develop that impairs the firm attachment of the prosthesis. The fact that surgeons can adapt to tissue conditions is also emphasized by the findings of other authors [12]. They performed live intraoperative measurements in sheep using pressure transducers. However, during mitral annuloplasty, their knotting forces averaged 2.7 ± 1.4 N, which is only one-fifth of the maximum force we determined for aortic valve implantation. Using tourniquets instead reduces the required forces to less than one tenth [7]. This entails a much higher safety margin and protects against tissue rupture as subsequent knotting can be done with much less effort.

**Figure 5:**
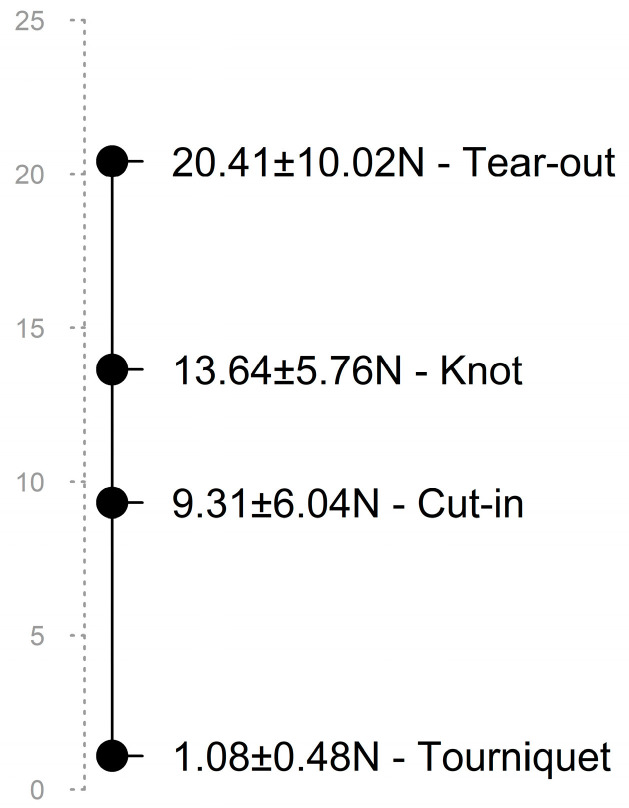
Virtual scale with all average force levels taken from the current and our previous study [7]. Even if the comparison is possible only to a limited extent, it can be seen that the tear-out force is far apart from the forces exerted by knots or tourniquets and unlikely to be reached. However, tying manual knots implies peak forces that exceed or come close to the cut-in threshold. In contrast, tightening of a tourniquet is least likely to cause damage due to its low strength.

All force levels from our current and the previous study [7] are depicted in Figure 5, united on a virtual scale. Although different experimental designs were used in either of our studies, all traction forces were determined perpendicular to the annulus with the same force gauge.

We could show that the right coronary portion of the annulus is more susceptible to tissue lesions than the remaining regions. This can occasionally be observed in the operating room as well. Collagen density and alignment may play a role in tissue tear resistance [9,10]. The aortic valve is located within the fibrous skeleton of the heart and stands in continuity with the mitral valve. The left and noncoronary aspects of the annular circumference are reinforced by the left and right fibrous trigones, respectively. In contrast, part of the right coronary annulus is in close proximity to the membranous septum. In the porcine heart, the extent of fibrous support at the aorto-mitral continuity is even weaker than in humans, as three quarters of the circumference are supported by the muscular ventricular septum [14]. Overall, the inhomogeneous anatomic features of the aortic annulus may explain the different resistance to suture tearing.

In the present study, we have determined critical force values for the aortic annulus depending on the location. This provides a kind of baseline reference that did not exist before. Together with the force values for knots and tourniquets from our previous study, conditions at the aortic annulus can be better assessed. First, we have learned that tying knots must be done very carefully as this occurs in close proximity to the critical cut-in range. Second, this appears to be particularly true along the right coronary portion of the annulus. From a theoretical point of view, this could also apply to the entire annulus when friable, calcified, or endocarditic tissue is present. Third, the use of tourniquets for temporary attachment of the prosthesis seems reasonable as the force values are at the lower end of the scale. Thus, the “Caput medusae” method offers a greater safety margin, provided that the subsequent knots are tied much more gently compared with primary knotting.

Our results could also be useful in the development of automatic knotting devices. In the future, such devices could have a calibrated scale, indicating the applied force at any given time. In addition, an adjustable force limit could be incorporated, similar to a torque wrench. In this way, the force could be individually preselected according to the particular tissue conditions to avoid annular rupture. It is also conceivable that devices could be developed to allow surgeons to assess their own tying forces to provide them with more refined tactile feedback. After all, it will always be the surgeon who adapts his/her actions to the patient based on personal experience and who must also accept failures in the process. As an aphorism attributed to Kerr L. White (1917–2014) states, “Good judgement comes from experience; experience comes from bad judgement”.

### Limitations

The work was conducted as a pilot ex vivo study in non-diseased porcine hearts. The transferability to a human heart in vivo may therefore be limited. The number of specimens was low and the measuring conditions were not entirely realistic. In particular, for visibility reasons, we did not use an abutment such as the suture ring but performed the measurements in free space instead. This led to improper distortions of the tissue. Furthermore, two of the four forces shown in Figure 5 came from a different experimental setup [7]. In the next step, measurements on a heart model with friable tissue are desirable.

## 5. Conclusions

In a porcine heart model, we showed that even in healthy tissue, a moderate average force of 9.31 N can reach a critical level where the Teflon™ pledget cuts into the annulus, while the more distant level, at which a complete tear-out occurs (20.41 N), is unlikely to be reached. The right coronary portion showed markedly lower threshold forces than the remaining regions of the annulus. As known from previous studies, rubber tourniquets generate lower forces than manual knots and thus may provide a higher safety margin against tissue rupture. From a theoretical aspect they are preferable, especially in the case of friable or infected tissue. Special care should always be taken in the right coronary area, which appears more susceptible to tissue damage.

## Figures and Tables

**Table 1:** Average “cut-in” and “tear-out” forces in relation to the anatomic position of the suture.

		Cut-In Forces		Tear-Out Forces	
Position	Unit	Mean ± SD	Median	Mean ± SD	Median
Right coronary	(N)	4.77 ± 3.28	3.61	10.67 ± 4.04	9.34
Left coronary	(N)	10.59 ± 5.89	9.93	26.60 ± 11.41	25.04
Noncoronary	(N)	11.19 ± 5.77	11.92	21.09 ± 6.82	22.02
Commissural	(N)	10.24 ± 6.86	9.17	22.03 ± 9.02	23.79

The mean and median values of the cut-in and tear-out forces are displayed separately according to the respective suture position. N, Newton; SD, standard deviation.

**Table 2:** Results of the univariable two-factorial ANOVA (Analysis of variance).

		Type III Sum of Squares	df	Mean Square	F	*p* Value
Dependent Variable: Cut-In Force						
**Position**	Hypothesis	320.007	3	106.669	3.091	0.040
	Error	1207.958	35	34.513		
**Heart**	Hypothesis	326.727	13	25.133	0.728	0.724
	Error	1207.958	35	34.513		
**Dependent Variable: Tear-Out Force**						
**Position**	Hypothesis	1620.646	3	540.215	7.594	<0.001
	Error	2703.249	38	71.138		
**Heart**	Hypothesis	782.886	13	60.222	0.847	0.611
	Error	2703.249	38	71.138		

Shown are the main effects of the fixed factor “Position” (left/right/noncoronary or commissural) and the random factor “Heart”, including the error variances. **Upper half**, ANOVA for cut-in force; **Lower half**, ANOVA for tear-out force. In both cases, the anatomic position has a significant effect on the threshold force, whereas the heart itself has no effect.

## Data Availability

The data underlying this article are available in the article and in its online Supplementary Materials.

## Supplementary Materials

The following supporting information can be downloaded at: https://www.mdpi.com/article/10.3390/medicina58081055/s1, Table S1: Raw Data.
